# Supplementary material for: A trajectory data compression algorithm based on spatio-temporal characteristics
Source: PeerJ Comput Sci. 2022 Oct 3;8:e1112. doi: 10.7717/peerj-cs.1112 (PMC9575842; doi:10.7717/peerj-cs.1112)
Supplement: Supplemental Information 1 [file peerj-cs-08-1112-s001.docx]

#include <iostream>

#include <cmath>

using namespace std;

struct gps//定义一种gps点结构 Define a GPS point structure

{

double lon;//经度 longitude

double lat;//纬度 latitude

char t[20];//时间 time

double time;//时间差 time difference

double speed;//速度 velocity

double angle; //方位角 azimuth

};

gps p[25000000];//存放输入的gps数据 Store the input GPS data

gps q[25000000];//存放输出的gps数据 Store the output GPS data

gps a;//a-e用于存放中间数据 a-e is used to store intermediate data

gps b;

gps c;

gps d;

gps e;

void input()//输入数据 input data

{

cout << "Reading data to be compressed" << endl;

FILE *fp1=fopen("before.txt","r");

for(int i = 0; ; i++){

fscanf(fp1,"%lf%lf%s%lf%lf%lf",&p[i].lat,&p[i].lon,&p[i].t,&p[i].time,&p[i].speed,&p[i].angle);

if(p[i].lat ==0)break;}

}

void initial()

{

a=p[0];

b=p[1];

c=p[2];

d=p[3];

e=p[4];

}

void compress()//压缩 compress

{

int j=0;

int x,y,z;

for(int i=5; ; i++){

if(p[i].lat ==0)break;

if(fabs(c.angle-b.angle)<180){

x=fabs(c.angle-b.angle);

}

else{

x=360-fabs(c.angle-b.angle);}

if(fabs(c.angle-d.angle)<180){

y=fabs(c.angle-d.angle);

}

else{

y=360-fabs(c.angle-d.angle);}

if(fabs(d.angle-a.angle)<180){

z=fabs(d.angle-a.angle);

} //z是为防止连续小转弯而设置的参数，在本版本并未启用，有待后续验证 Z is a parameter set to prevent continuous small turns. It is not enabled in this version and needs to be verified later

else{

z=360-fabs(d.angle-a.angle);}

if(c.time<10 && fabs(c.speed-b.speed)<b.speed*0.5 && fabs(c.speed-d.speed)<d.speed*0.5 && x<8 && y<8 )//这表示取T1=8°，T2=50% That means T1 is equal to 8°, T2 is equal to 50%

{

c=d;

d=e;

e=p[i];}

else{

q[j]=a;

a=b;

b=c;

c=d;

d=e;

e=p[i];

j++;}

}

q[j]=d;

q[++j]=e;}

void write()//将压缩后的数据写入文件compress.txt Writes compressed data to a file

{

FILE *fp2=fopen("compress.txt","w");

printf("The compression result is:\n");

for(int j = 0; ; j++){

if(q[j].lat ==0)break;

printf("%.5lf %.5lf %s\n",q[j].lat,q[j].lon,q[j].t);

fprintf(fp2,"%.5lf %.5lf %s\n",q[j].lat,q[j].lon,q[j].t);

}

fclose(fp2);}

int main()

{

input();

initial();

compress();

write();

system("pause");

return 0;}
